# Slightly increased level of DNA migration in the comet assay: does statistical significance equal biological significance?

**DOI:** 10.1093/mutage/geaf004

**Published:** 2025-02-18

**Authors:** Peter Møller, Andrew Collins, Adriana Rodriguez-Garraus, Sabine A S Langie, Roger Godschalk, Amaya Azqueta

**Affiliations:** Department of Public Health, Section of Environmental Health, University of Copenhagen, Øster Farimagsgade 5A, DK-1014 Copenhagen K, Denmark; Department of Nutrition, University of Oslo, Oslo, Norway; NorGenotech AS, Oslo, Norway; Department of Pharmaceutical Science, School of Pharmacy and Nutrition, University of Navarra, C/Irunlarrea 1, 31009 Pamplona, Spain; Department of Pharmacology & Toxicology, School for Nutrition and Translational Research in Metabolism (NUTRIM), Maastricht University, Maastricht, The Netherlands; Department of Pharmacology & Toxicology, School for Nutrition and Translational Research in Metabolism (NUTRIM), Maastricht University, Maastricht, The Netherlands; Department of Pharmaceutical Science, School of Pharmacy and Nutrition, University of Navarra, C/Irunlarrea 1, 31009 Pamplona, Spain

**Keywords:** comet assay, statistical significance, biological significance, DNA damage, DNA migration

## Abstract

In the comet assay, DNA damage is assessed by differences in DNA migration from gel-embedded nucleoids. Even a small difference in DNA migration between exposure groups can be statistically significant but may invite speculation about the biological significance of such slight increases in DNA migration. A small difference can be defined as a net difference of 1–2% Tail DNA, but background levels of DNA migration typically vary already more than 1–2% Tail DNA between studies. Here, we have used studies on ionizing radiation to assess the lowest detectable differences in DNA migration; variation in exposure-effect relationships; variation in central tendencies of DNA migration; unsystematic (residual) variation; and the actual number of lesions detectable with the comet assay. A total of 51 studies on ionizing radiation exposure in mammalian cells have been systematically reviewed, including results from ring-trial studies where the same batch of irradiated cells has been analysed in different laboratories. Ring-trial studies have shown that unsystematic variation is approximately 4% Tail DNA in studies on ionizing radiation. Studies on ionizing radiation in cell cultures have shown statistically significant effects when the net increase of DNA migration is 0.3–3.1% Tail DNA. Among those experiments, the ones with optimal assay conditions to detect low levels of DNA damage show statistically significant effects with doses of around 0.30 Gy, which corresponds to approximately 350 lesions per diploid cell. However, it has also been shown that the same dose of ionizing radiation can give rise to different levels of DNA migration (i.e. 0.7–7.8% Tail DNA per Gy) in different studies. In summary, the results show that even a small statistically significant difference in DNA migration has biological significance within the same experiment, but comparisons of DNA migration values between studies have limited biological implications.

## Introduction

The comet assay is a sensitive technique for the detection of DNA damage, meaning that it can detect increased levels of genotoxicity at low doses of DNA-damaging agents. However, it is important to understand that the comet assay does not detect DNA damage per se but is based on the measurement of DNA migration in agarose gels resulting from breaks in DNA strands. The most used primary comet descriptor is the percentage of DNA in the comet tail (%Tail DNA) [1]. By definition, the range is between 0% and 100% Tail DNA. A slight increase in DNA migration of, for instance, 1 or 2% Tail DNA might well be seen between unexposed and exposed cells, of animals or humans. For the comet assay investigator, such a small difference will not be apparent when viewing individual comets but will appear as a statistically significant effect in data analysis. Thus, it seems important to explore the biological significance of differences in DNA migration levels, which are so small that it requires statistical tools to ascertain a difference between exposure groups.

The aim of this review is to discuss the biological significance of small differences in DNA migration values in the comet assay. In the paper, we distinguish between *DNA migration* and *DNA damage*. The latter refers to the actual level of lesions in DNA. A genotoxic agent causes *DNA damage*, which can be expressed as a frequency (e.g. lesions per million base pairs), whereas *DNA migration* is a relative measurement specific to the comet assay. In the comet assay, *DNA migration* is measured by different comet descriptors such as tail length, %Tail DNA, tail moment (the product of tail length and %Tail DNA), and visual score (a manual scoring system where comets are sorted into different classes depending on their form). A sample with a known level of *DNA damage* can give rise to different *DNA migration* values by analysing it in different assay conditions. Therefore, *DNA migration* values in different experiments only correspond to the same *DNA damage* levels when the assay conditions are identical.

The review introduces, first, concepts of statistical and biological significance in general, and then in the context of the comet assay. Results from ionizing radiation experiments in mammalian cell cultures are used for discussion of the biological significance of small differences in DNA migration levels in the comet assay. We use the terms *biological significance* and *statistical significance* to emphasize the two interpretations of significance in studies where exposure only causes small differences in DNA migration levels.

## Statistical vs biological significance

Statistical significance is easy to define (normally with the threshold of *P* < .05), but biological significance is a somewhat more uncertain term. The European Food Safety Authority has considered that *‘a biologically relevant effect can be defined as an effect considered by expert judgement as important and meaningful for human, animal, plant or environmental health’* and further clarifies that *‘it therefore implies a change that may alter how decisions for a specific problem are taken’* [2]. It goes without saying that the aim of the present review comes with the premise that comet assay results have important biological implications. This premise is easily challenged because most DNA lesions that can be detected by the comet assay are repaired relatively efficiently and quickly. Thus, the relevance of the comet assay as a genotoxicity test rests on the assumption that a minor proportion of the lesions are not repaired and give rise to mutations and/or chromosome damage during DNA replication.

We have tied both statistical and biological effects to the term *significance*. This is done to emphasize the connection between the results of statistical analyses and the biological effect. For the biological effect *relevance* and *importance* are used as synonyms for *significance* [2]. Interestingly, the meaning of *statistical significance* is generally clear to biologists (i.e. there is a difference between two values, with *P* < .05), whereas statisticians tend to have a nuanced view on the interpretation of statistical test results [3]. It is important to note the distinction between biological and statistical significance because relatively large biological effects tend to be regarded as irrelevant if they are not statistically significant, whereas statistically significant effects tend to be regarded as relevant even if the effect size is small. It is our experience that questions about the biological significance of low levels of DNA migration values in the comet assay typically come up during informal discussions among researchers. Reviewers may also dare to ask such questions, which are difficult to answer in a satisfactory way and the claim that statistically significant differences are biologically relevant may not be a convincing reason.

In order to make the setting of the review more tangible, we provide here an example of a clash between statistical and biological significance. It is relatively rare that several *in vivo* studies on weakly genotoxic agents are done in the same laboratory. However, an example of this research exists, with pulmonary exposure to carbon black nanomaterials; this agent is classified as possibly carcinogenic to humans (IARC Group 2B), based mainly on experimental evidence in rats and mechanistic evidence of oxidative stress, inflammation, and genotoxicity [4]. Nanosized carbon black exposure generates DNA strand breaks in cell culture experiments [5]. For many years, one laboratory has used carbon black as a positive control for pulmonary inflammation, and the comet assay has been used in a battery of tests to assess genotoxicity in lung tissue [6]. The experimental evidence comprises genotoxicity data from more than 800 mice, reported in 14 different publications. Interestingly, only two of the 14 studies have shown statistically significant increases in DNA strand breaks (i.e. % Tail DNA) in lung tissue, whereas a pooled analysis of results shows a net increase of 0.9% Tail DNA (95% CI: −0.1, 1.9) and 0.8% Tail DNA (95% CI: 0.1, 1.5) at days 1 and 3 post-exposure, respectively [6]. It seems fair to consider the modest increase in DNA strand breaks as a genotoxic mechanism of action, which required considerable statistical power to reveal as statistically significant. However, the nagging question is whether this observation of slightly increased DNA migration in lung tissue really has any biological significance when it requires 800 mice to obtain an effect that is barely statistically significant, and furthermore appears statistically significant in just two studies.

## Assessment of biological significance in the comet assay

**Figure 1.**
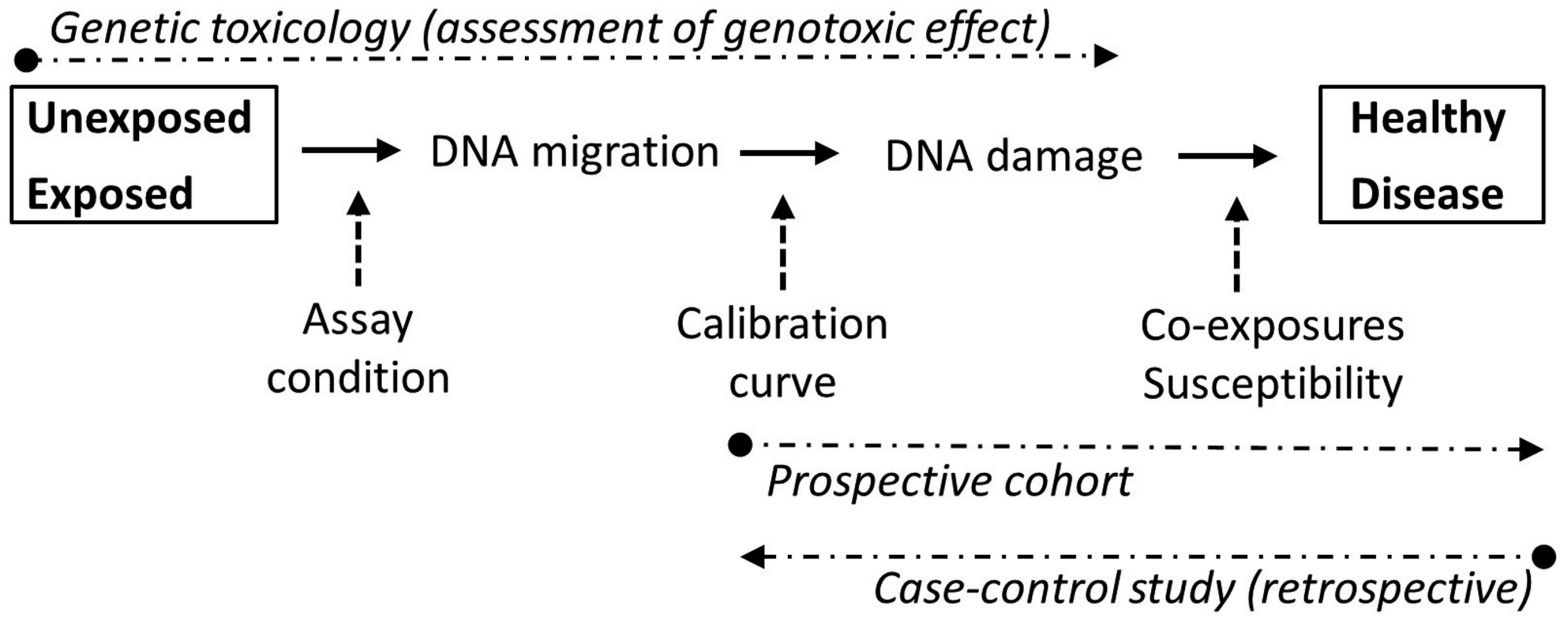
Approaches to investigate the biological significance in DNA migration values in the comet assay.

An understanding of the biological significance of small differences in DNA migration values in the comet assay can be obtained by two distinct approaches (outlined in Fig. 1). The biological significance of comet assay results can be assessed as the predictive value for disease outcomes. This can be assessed in prospective cohort studies, although it requires a large number of subjects to control for competing risk factors and to obtain statistical power to detect relatively rare outcomes such as cancer. This is illustrated by results from a recently published prospective study on pooled data from different biomonitoring studies where high levels of DNA migration in leukocytes were associated with increased overall mortality in the follow-up period, whereas there were too few cases of tissue-specific tumours to obtain robust associations with cancer outcomes [7,8]. Thus, biobank-based retrospective nested case-control studies (right-to-left direction in Fig. 1) are more feasible to obtain information about the association between DNA damage levels and risk of disease in humans. Unfortunately, comet assay results from biobank-based nested case-control studies have not been published.

Another approach (left-to-right in Fig. 1) assesses how large the difference in DNA migration between experimental groups is, relative to the random variation. The level of DNA migration depends on the conditions used in some of the steps of the comet assay and therefore, a direct comparison of primary comet descriptors is not optimal for the assessment of biological significance. It is more informative to calibrate the comet assay with ionizing radiation to transform primary comet descriptors to lesions per million base pairs (see the supplement for a detailed description of calibration in the comet assay). This manoeuvre provides information on the actual frequency of lesions in DNA.

## The use of ionizing radiation to study biological significance

Studies on ionizing radiation have been instrumental in the assessment of the biological significance of DNA migration levels in the comet assay because the dose at the target molecule (i.e. DNA) is easy to control by the time of exposure. We refer to the exposure as *genotoxic dose,* because there is a proportionality between the magnitude of exposure (measured in Gy) and the level of DNA damage. The characteristics of ionizing radiation and its biological effects are briefly described in the Supplementary Material, abstracted mainly from extensive reviews on the topic [9,10].

Ionizing radiation is an ideal agent to assess the lowest effective genotoxic dose and variation between studies in genotoxic effects, and to calibrate the comet assay (i.e. convert primary comet descriptors to frequency of lesions). Below we use studies on ionizing radiation to determine the lowest effective genotoxic dose to increase DNA migration and also to assess experimental variation in the comet assay. The studies on ionizing radiation have been identified by a systematic literature review (described in detail in the Supplementary Material). In brief, we have included *in vitro* studies on mammalian cells exposed to hard gamma rays or X-rays (i.e. studies on soft X-rays are not included as they have relatively low energy). Inclusion criteria for studies in the review were: use of the alkaline version of the comet assay; sufficient description of the experimental procedure; and use of more than one dose of ionizing radiation in mammalian cells.

### Low-dose exposure to ionizing radiation; lowest effective genotoxic dose to increase DNA migration

Studies on the effect of ionizing radiation span the entire history of the comet assay from the 1980s to the present day. In this review, low-dose ionizing radiation (i.e. ≤ 1 Gy) studies are useful for the assessment of the lowest effective dose of genotoxicity to detect a statistically significant increase in DNA migration. Unfortunately, early studies tended to use tail length and tail moment as primary comet descriptors, which have been increasingly replaced by %Tail DNA and to some extent visual score.

Table 1 summarizes exposure conditions and relevant comet assay details in studies that have assessed DNA migration in cells after exposure to doses of ionizing radiation up to 1 Gy. The duration of electrophoresis is typically longer in these studies; the median period of electrophoresis is 30 min in the studies that have investigated low-dose exposure to ionizing radiation (Table 1) as compared to a median time of 20 min in high-dose studies (Table 2). Due to the heterogeneity of comet descriptors (tail length, tail moment, visual score, comet length, and %Tail DNA), it is only possible to assess the doses of ionizing radiation that have yielded a statistically significant increase in DNA migration. Eight studies have shown statistically significant increased DNA migration at 0.05-0.30 Gy [12,14–16,18,22,23,25]. An overall analysis indicates that 0.3 Gy is the lowest dose where all studies show an increased level of DNA migration, whereas 0.25 Gy is the highest dose with inconsistent results (i.e. some studies have shown increased DNA migration at 0.25 Gy, whereas other studies have shown unaltered levels of DNA migration). There is a higher proportion of positive results at doses of 0.25 Gy or higher (57 statistically significant effects out of 59 results at doses ≥ 0.25 Gy as compared to 22 statistically significant effects out of 28 results at doses lower than 0.25 Gy in the 21 studies in Table 1, χ^2^ = 7.4, *P* < .01). Studies that have assessed DNA migration by %Tail DNA indicate that the net increase at the lowest dose with statistical significance is 0.8-3.1 %Tail DNA (mean = 2.1% Tail DNA, standard deviation = 0.8% Tail DNA, *n* = 6) [25–27,29–31].

**Table 1.** DNA migration in mammalian cells by low-dose exposure to ionizing radiation (≤ 1 Gy)

Reference	Cell type (species)^a^	Radiation source	Doses (cGy)^b^	Key assay conditions^c^	Comet descriptor^d^
Singh *et al*., 1988 [11]	Lymphocytes (human)	X-rays (0.2 Gy/min)	**25**, **50**, **100**	0.5% NR (25 V) 20 min	Tail length
Vijayalaxmi *et al*., 1992 [12]	Lymphocytes (human)	X-rays (0.4 Gy/min)	1.3, 2.6, **4.9**, **10**, **25**, **50**, **100**	0.5% NR (25 V) 40 min^e^	Tail length
Vijayalaxmi *et al*., 1993 [13]	Lymphocytes (human)	X-rays (0.4 Gy/min)	**5**, **10**, **25**, **50**	0.5% NR (25 V) 40 min	Tail length
Singh *et al*., 1994 [14]	Lymphocytes (human)	X-rays (1 Gy/min)	**3.2**, **6.4**, **12.8**, **25.6**	0.9% NR (25 V) 60 min	Tail length
Plappert *et al*., 1995 [15]	Leukocytes (human)	X-rays (0.7 Gy/min)	**5**, **10**, **25**, **50**, **75**, **100**	0.5% 0.8 V/cm 30 min	Tail moment
Malyapa *et al*., 1998 [16]	Lymphocytes (rat)	Gamma rays (1.0 Gy/min by ^137^Cs decay)	**0.6**, **1**, **3**, **5**	0.6% 0.6 V/cm 25 min	Tail length and tail moment
Gajendiran *et al*., 2000 [17]	Lymphocytes (human)	Gamma rays (≤ 0.1 Gy/min by ^60^Co decay)	**12.5**, **25**, **50**, **100**	0.8% 0.8 V/cm 20 min	Tail moment
He *et al*., 2000 [18]	Lymphocytes (human)	X-rays (dose rate not reported)	2, **5**, **10**, **25**, **50**, **100**	0.4% NR (25 V) 20 min	Comet length
Nascimento *et al*., 2001 [19]	Leukocytes (human)	Gamma rays (0.7 Gy/min by ^60^Co decay)	**20**, **60**, **100**	0.5% NR (25 V) 30 min	Tail length
Wojewodzka *et al*., 2002 [20]	Ovary cells (CHO-K1, Chinese hamster)	X-rays (1.2 Gy/min)	25, **50**, **100**^f^	1.0% 1.2 V/cm 30 min	Tail moment
Gomolka *et al*., 2005 [21]	Lymphocytes (human)	X-rays (0.5, 0.75, 1, 1.5, 2 or 3 Gy; 0.42 Gy/min) or the same doses by ^137^Cs (0.66 Gy/min) or ^60^Co (0.42 Gy/min) decay	**50**, **75**, **100**	0.45% 0.8 V/cm 30 min	%Tail DNA (1.2% at 50 cGy)^g^
Masoomi *et al*., 2006 [22]	Lymphocytes (human)	Gamma rays (0.5 Gy/min by ^60^Co decay)	**30**, **100**	NR 0.56 V/cm 20 min	Visual score (5-class)
Sudprasert *et al*., 2006 [23]	Lymphocytes (human)	Gamma rays (0.2 Gy/min by ^137^Cs decay)	**5**, **10**, **20**, **50**	NR NR (24 V) 20 min	Tail moment
Wojewodzka *et al*., 2008 [24]	Lymphocytes (human)	X-rays (1.5 Gy/min)	**30**, **50**, **80**, **100**^h^	0.5% 1.2 V/cm 30 min	Tail moment
Kennedy *et al*., 2012 [25]	Lymphocytes (human)	Gamma rays (12 Gy/min ^60^Co decay)	10, **20**, **30**, **40**, **50**, **60**, **70**, **80**, **90**, **100**^i^	0.55% 1.1 V/cm 30 min	%Tail DNA (3.1% at 20 cGy)
Saini *et al*., 2012 [26]	Lymphocytes (human)	Gamma rays (0.7 Gy/min by ^60^Co decay)	**10**, **30**, **60**, **100**	0.45% NR (25 V) 20 min	%Tail DNA (0.8% at 10 cGy)
Bannik *et al*., 2013 [27]	Lymphocytes (mice)	Gamma rays (0.5 Gy/min by ^137^Cs decay)	25, **50**, **100**^j^	0.45% 0.8 V/cm 30 min	%Tail DNA (1.8% at 50 cGy)
Tepe Cam and Seyhan, 2013 [28]	Hair root cells (human)	Gamma rays (8.4 Gy/min by ^137^Cs decay)	**50**, **100**	0.5% NR (25V) 30 min	Tail length and tail moment
Gutzkow *et al*., 2013 [29]	Lymphocytes (human)	X-rays (3 or 10 Gy/min)^k^	10, **30**, **50**, **70**, **100**	0.75% 0.9 V/cm 30 min	%Tail DNA (2.5% at 50 cGy)
Toprani and Das, 2015 [30]	Lymphocytes (human)	Gamma rays (1.0 Gy/min by ^60^Co decay)	10, **30**, **60**, **100**	0.5% NR (25V) 20 min	%Tail DNA (2.2% Tail DNA at 30 cGy)
Panek *et al*., 2018 [31]	Lymphocytes (human)	X-rays (1.8 Gy/min)	**30, 50**, **75**, **100**	Not reported NR (30 V) 30 min	%Tail DNA (2.0% at 30 cGy)

^a^Cell types are reported as leukocytes if whole blood has been used. For simplicity, peripheral blood mononuclear cells and lymphocytes are reported as lymphocytes in the table.

^b^Doses causing a statistically significant effect on DNA migration are highlighted in bold text. The doses are reported in cGy (i.e. 100 cGy = 1 Gy). Doses higher than 100 cGy are not included in the table.

^c^Key assay conditions are defined as those that the researchers have altered to increase the sensitivity of the assay (i.e. lower concentration of low-melting point agarose and higher electrophoretic field strength). The information is the percentage of agarose (first line), electrophoretic strength (V/cm or only reported as voltage; second line) and time of electrophoresis (third line). In certain cases, the information is not reported (NR) in the paper.

^d^In cases where %DNA in tail has been used, we have highlighted the net increase (subtracting the negative control) and the dose of ionizing radiation.

^e^Experiments include different alkaline unwinding and electrophoresis times. The results in the table are based on 40 min electrophoresis.

^f^There is uncertainty about the lowest effective dose because it has been based on a visual inspection of the results in a figure in the publication.

^g^Statistical significance is not reported in the publication, but comparison of the lowest dose and controls indicate a statistical difference (paired Student’s test on mean %Tail DNA values by exposure to ionizing radiation from three different sources).

^h^Dose-response experiments were done on B- or T-lymphocytes. B-lymphocytes seemed more sensitive than T-lymphocytes to ionizing radiation (increased level of DNA damage at 300 and 500 cGy, respectively), although the sensitivity was not different over the whole dose range.

^i^Irradiation done on gel-embedded nucleoids.

^j^Statistical significance is not reported in the original publication. The difference in %Tail DNA between 0.5 Gy and 0 Gy (approximately 1.8% Tail DNA) is larger than the SEM of 0.5 Gy, indicating that 0.5 Gy has slightly increased the level of DNA migration as compared to the control.

^k^The reported statistics for the lowest dose (P = 0.017, Mann-Whitney U test) is not possible for group sizes of 3 independent tests. In addition, there are overlapping SEM intervals (i.e. proxy-measure for 95% confidence interval). We have used 30 cGy as lowest effective dose because there are non-overlapping SEM intervals.

**Table 2.** DNA migration in mammalian cells by high-dose exposure to ionizing radiation (≥ 1 Gy)

Reference	Cell type (species)^a^	Radiation source	Doses (Gy)	Key comet assay condition^b^	Dose response (%Tail DNA per Gy)
Panayiotidis *et al*., 2004 [32]	Lung epithelial cells (A549)	Gamma rays (2.2 Gy/min by ^137^Cs decay)	1, 2, 4, 8	NR 1 V/cm 30 min	2.1
Gomolka *et al*., 2005 [21]	Lymphocytes (human)	X-rays (0.42 Gy/min) and gamma rays (0.66 Gy/min by ^137^Cs or 0.42 Gy/min by ^60^Co decay)	0.5, 0.75, 1, 1.5, 2	0.45% 0.8 V/cm 30 min	2.7 (^137^Cs), 3.0 (^60^Co) and 2.7 (X-rays). No difference in generation of DNA strand breaks by between radiation sources
Kumaravel and Jha, 2006 [33]	Leukocytes (human)	Gamma rays (1.0 Gy/min by ^137^Cs decay)	1, 2, 4, 8	0.5% NR (25 V) 20 min	2.6 (mean of mean) and 2.9 (mean of medians)
Pitozzi *et al*., 2006 [34]	Lymphocytes (human) and melanoma cells (mouse)	Gamma rays (0.2 Gy/min by ^60^Co decay) and X-ray (2.0 Gy/min)	1.5, 2.5, 5, 7.5, 10 (gamma rays) and 1.3, 2, 2.5, 5 10 (X-rays)	1% 0.8 V/cm 20 min	2.4 (^60^Co), 2.8 (X-ray, lymphocytes) and 2.4 (X-rays, melanoma cells)^c^
Smith *et al*., 2006 [35]	Lymphoma cells (L5178Y, mouse)	Gamma rays (4.64 Gy/min by an unspecified isotope)	1, 5, 10	0.5% 0.7 V/cm 20 min	1.3
Giovanetti *et al*., 2008 [36]	Lymphocytes (mouse)	X-rays (1.1 Gy/min)	0.1, 1	0.5% 0.7 V/cm 40 min	5.8
Li *et al*., 2008 [37]	Lewis lung carcinoma cells (mouse)	X-rays (4.0 Gy/min)	0.5, 1, 2, 3, 4, 8, 12	0.56% 1 V/cm 25 min	2.2 (SEM: 0.2)
Vivek Kumar *et al*., 2009 [38]	Lymphocytes (human)	Gamma rays (1.6 Gy/min by ^60^Co decay)	0.5, 1, 2, 4, 7, 10	1% 0.7 V/cm 30 min	3.0^d^
Miklos *et al*., 2009 [39]	Lymphocytes (human)	Gamma rays (3.0 Gy/min by ^60^Co decay)^e^	0.1, 4	0.5% 1 V/cm 20 min	5.0
Wu *et al*., 2009 [40]	Keratinocytes (HaCaT, human)	Gamma rays (3.0 Gy/min by ^137^Cs decay)	3, 6, 9, 12, 15	0.4% 0.85 V/cm 30 min	1.9
Forchhammer *et al*., 2010 [41]	Monocytes (THP-1, human)	Gamma rays (3.8 Gy/min by ^137^Cs decay)	2.5, 5, 10	0.6-1.0% 0.8-1.6 V/cm 20-30 min	5.7 (SD: 2.4, Min: 0.7, Max: 8.3, results from 11 different laboratories)
Johansson *et al*., 2010 [42]	Monocytes (THP-1, human)	Gamma rays (3.8 Gy/min by ^137^Cs decay)	2.5, 5, 10	0.6-2.0% 0.6-1.5 V/cm 20-30 min	5.2 (SD: 2.3, Min: 0.9, Max: 8.0; results from 9 different laboratories)
Ersson and Möller, 2011 [43]	Lymphocytes (human)	Gamma rays (3.8 Gy/min by ^137^Cs decay)	2.5, 5, 7.5	0.7% 0.7-1.6 V/cm 20 or 30 min	4.1 (20 min/1.15 V/cm), 5.7 (30 min/1.15 V/cm), 3.5 (20 min/0.7 V/cm), 6.3 (20 min/1.15 V/cm) and 8.9 (20 min/1.6 V/cm)
Guerci *et al*., 2011 [44]	Lymphocytes (human)	Gamma rays (6.0 Gy/min by ^137^Cs decay)	1, 2, 4, 6, 8	0.45% NR (25 V) 20 min	5.0
Forchhammer *et al*., 2012 [45]	Lymphocytes (human)	Gamma rays (3.8 Gy/min by ^137^Cs decay)	2.5, 5, 7.5	0.61% 0.7-1.7 V/cm 20-30 min	4.4 (SD:1.8, Min: 1.5, Max: 6.4, results from 9 different laboratories)^f^
Kennedy *et al*., 2012 [25]	Lymphocytes (human)	Gamma rays (12 Gy/min ^60^Co decay)	1, 2, 3, 4, 5, 6, 7, 8	0.55% 1.1 V/cm 15 min	4.7^g^
Seidel *et al*., 2012 [46]	Leukocytes (human)	X-rays (2.1 Gy/min)	2, 4, 8	0.5% 0.8 V/cm 30 min	4.6
Bannik *et al*., 2013 [27]	Lymphocytes (mice)	Gamma rays (0.5 Gy/min by ^137^Cs decay)	0.3, 0.25, 0.5, 1, 2	0.45% 0.8 V/cm 30 min	4.9
Ersson *et al*., 2013 [47]	Lymphocytes (human)	Gamma rays (3.8 Gy/min by ^137^Cs decay)	2.5, 5, 7.5	0.5-1.0% 0.7-1.7 V/cm 20-30 min	4.6 (SD: 2.3, Min: 2.0, Max: 8.4, results from 10 different laboratories)
Godschalk *et al*., 2013 [48]	Monocytes (THP-1, human)	Gamma rays (3.8 Gy/min by ^137^Cs decay)	2.5, 5, 10	0.6-1.0% 0.6-1.3 V/cm 20-30 min	6.0 (SD: 2.2, Min: 2.4, Max: 8.5; results from 7 different laboratories)
Gutzkow *et al*., 2013 [29]	Lymphocytes (human)	X-rays (3 or 10 Gy/min)	1, 3, 5, 8, 10, 15	0.75% 0.9 V/cm 20 min	3.8 (glass slide) or 4.5 (mini-gel film)^h^
Godschalk *et al*., 2014 [49]	Lymphocytes (human)	Gamma rays (3.8 Gy/min by ^137^Cs decay)	2.5, 5, 7.5	0.5-1.0% 0.7-1.7 V/cm 20-30 min	4.4 (SD: 2.3, Min: 1.8, Max: 9.0, results from 12 different laboratories)
Osipov *et al*., 2014 [50]	Lymphocytes (human)	X-rays (0.85 Gy/min)	1, 2, 3, 4, 5, 6	0.75% 0.75 V/cm 20 min	6.5
Sirota *et al*., 2014 [51]	Bone marrow cells (mouse)	X-rays (1.1 Gy/min)	3, 5, 8	0.9% 2.0 V/cm 20 min	4.9^i^
Toprani and Das, 2015 [30]	Lymphocytes (human)	Gamma rays (1.0 Gy/min by ^60^Co decay)	0.1, 0.3, 0.6, 1, 2	0.5% NR (25V) 20 min	5.1
Enciso *et al*., 2018 [52]	Lymphoblasts (TK6, human)	X-rays (3.8 Gy/min)	2, 4, 6, 8, 10, 12	0.75% 0.85 V/cm 25 min	5.0^j^
Hansen *et al*., 2018 [53]	Lymphocytes (human)	X-rays (1.0 Gy/min)	6, 10	0.75% 0.8 V/cm 20 min	5.2 (mean of 1 h and overnight lysis)
Koppen *et al*., 2018 [54]	Leukocytes (human)	Unknown gamma radiation source	1, 3, 5, 8, 10, 15	0.8% 1 V/cm 20 min	6.6
Sirota *et al*., 2028 [55]	Leukocytes (human) and splenocytes (mice)	X-rays (1.1 Gy/min)	2, 4, 8	1% 2 V/cm 20 min	2.0
Cassano *et al*., 2020 [56]	Lung epithelial cells (A549, human)	X-rays (0.5 Gy/min)^k^	1, 2, 4, 8	0.4% 1.0 V/cm 20 min	4.0
Andersen *et al*., 2021 [57]	Monocytes (THP-1, human)	Gamma rays (1.0 Gy/min by ^137^Cs decay)^l^	2.5, 5, 7.5, 10	0.7% 1.15 V/cm 25 min	2.8
Roman *et al*., 2021 [58]	Prostate adenocarcinoma cells (PC-3, human)	X-ray (2.1 Gy/min)	2, 4, 6, 8, 10	0.5% 0.7 V/cm 30 min	3.9
Harnung Scholten *et al*., 2021 [59]	Monocytes (THP-1, human)	Gamma rays (1.0 Gy/min by ^137^Cs decay)	2.5, 5, 7.5, 10	0.7% 0.83 V/cm 25 min	5.1
Møller, 2022 [60]	Monocytes (THP-1, human)	Gamma rays (1.0 Gy/min by ^137^Cs decay)	2.5, 5, 7.5, 10	0.7% 0.83 V/cm 25 min	4.1
Brunborg *et al*., 2023 [61]	Lymphocytes or leukocytes (human)^m^	Gamma and X-rays (1.1-6 Gy/min)	1, 3, 5, 8, 10, 15	0.7% 0.8 V/cm 20 min	4.9 (standard protocol) and 3.0-6.1 (favourite protocol)

^a^Cell types are reported as leukocytes if whole blood has been used. For simplicity, peripheral blood mononuclear cells and lymphocytes are reported as lymphocytes in the table.

^b^Key assay conditions are defined as those that the researchers have altered to increase the sensitivity of the assay (i.e. lower concentration of low-melting point agarose and higher electrophoretic field strength). The information is the percentage of agarose (first line), electrophoretic strength (V/cm or only reported as voltage; second line) and time of electrophoresis (third line). In certain cases, the information is not reported (NR) in the paper.

^c^No difference in slopes between sources of ionizing radiation.

^d^Mean slope of lysis protocols (i.e. strong alkali deprotonization versus high-salt detergent lysis condition).

^e^Information on radiation equipment has been collected from a previous publication by the authors [62].

^f^Mean slope with own protocol was 4.7 (SD: 1.7) %Tail DNA per Gy. Based on results from 10 laboratories.

^g^Irradiation done on lysed gel-embedded nucleoids.

^h^Results are based on irradiations in suspension.

^i^Slides were processed in two laboratories with electrophoresis at ‘low’ (start: 6°C, end: 14°C) and ‘high’ temperature (start: 15°C, end: 20°C). The slope is the mean of dose-response relationships in the two laboratories. Highest dose (8 Gy) is not included.

^j^The dose-response relationship (slope) is reported to be ‘9.8183’ in the paper. However, 12 Gy induces approximately 60% Tail DNA (i.e. slope = 5% Tail DNA per Gy).

^k^The dose-response relationship seems to be log-linear rather than linear over the total dose range (1, 2, 4, 8 Gy). We have restricted the dose-relationship to 0-2 Gy.

^l^Doses of 7.5 and 10 Gy have been omitted due to non-linearity.

^m^Results come from a ring trial with eight laboratories that assessed calibration curves by either a laboratory-specific (favourite) or standard protocol (reported in the table). The results are mean values from ionizing radiation experiments on cells in suspension or on gel-embedded cells from five laboratories that used image analysis and reported key comet procedure information.

Collectively, using conditions with increased sensitivity indicates consistency between studies at 0.30 Gy for statistically significant increased level of DNA migration in the comet assay. However, this is a conservative estimate and the lower limit of the dose for statistical significance may be as low as 0.05 Gy, as demonstrated in a number of studies [12–16,18,23].

### High-dose exposure to ionizing radiation; variation in DNA migration in samples exposed to ionizing radiation

**Figure 2.**
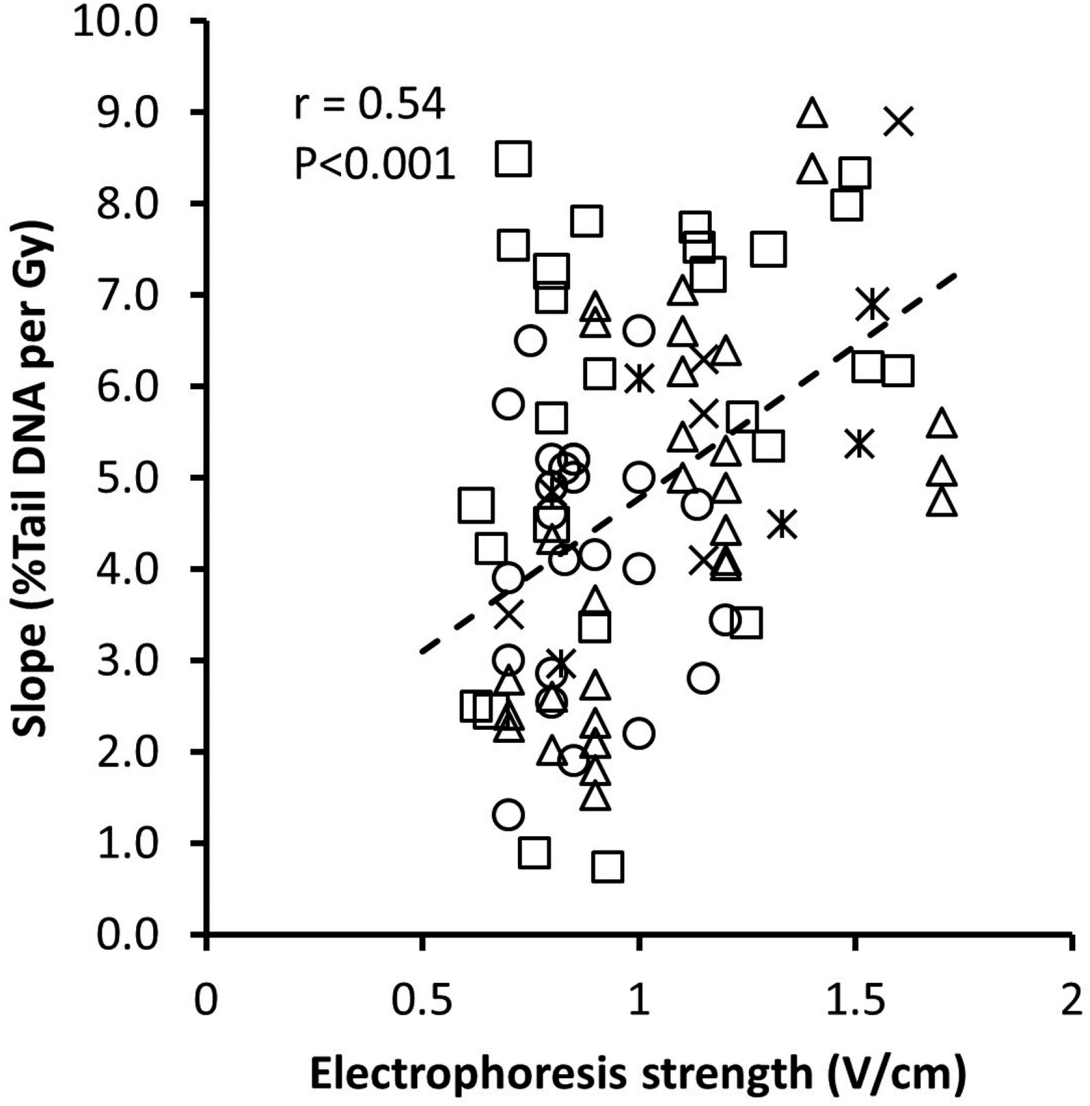
Correlation between voltage gradient (V/cm) and DNA migration (%Tail DNA) in cells exposed to ionizing radiation. Results from European Comet Assay Validation Group (ECVAG) ring trial samples are segregated into THP-1 cells exposed to 0-10 Gy (squares) [41,42,48] or peripheral blood mononuclear exposed to 0-7.5 Gy (triangles) [45,47,49]. One laboratory has assessed the dose-response relationship in ECVAG samples by using different electrophoresis conditions (crosses) [43]. One study has assessed the slope of ionizing radiation calibration curves in different laboratories (double crosses) [61]. A number of other laboratories have used different types of cells for ionizing radiation experiments (circles; different cell types are not shown in the graph). The line shows the relationship between voltage gradient and the dose-response relationship (slope) in samples after exposure to ionizing radiation (P < 0.001, linear regression).

Table 2 summarizes the information from studies that have assessed the relationship between high doses of ionizing radiation (i.e. ≥ 1 Gy) and DNA migration as %Tail DNA in the standard comet assay, the highest doses tested being 10-15 Gy. Differing comet assay conditions applied to these studies. Statistical analysis of the relationship between comet assay conditions and induction of DNA strand breaks (i.e. %Tail DNA increase per Gy) indicates only an effect of the electric potential (V/cm, *P* < .001, linear mixed effect model with agarose density, voltage gradient and time as continuous variables and study as categorical (absorbed factor)). Figure 2 depicts the relationship between the voltage gradient of electrophoresis and the induction of DNA migration. While there is a strong statistical relationship, dose–response relationships between different studies vary widely; for example, the induction of DNA migration varies from 0.7 to 7.8% Tail DNA per Gy at a voltage gradient of 0.9–1.1 V/cm. Thus, a net increase of 0.7% (i.e. slightly increased level of DNA migration) and 7.8% are both regarded as equivalent to the number of lesions generated by 1 Gy of ionizing radiation.

**Figure 3.**
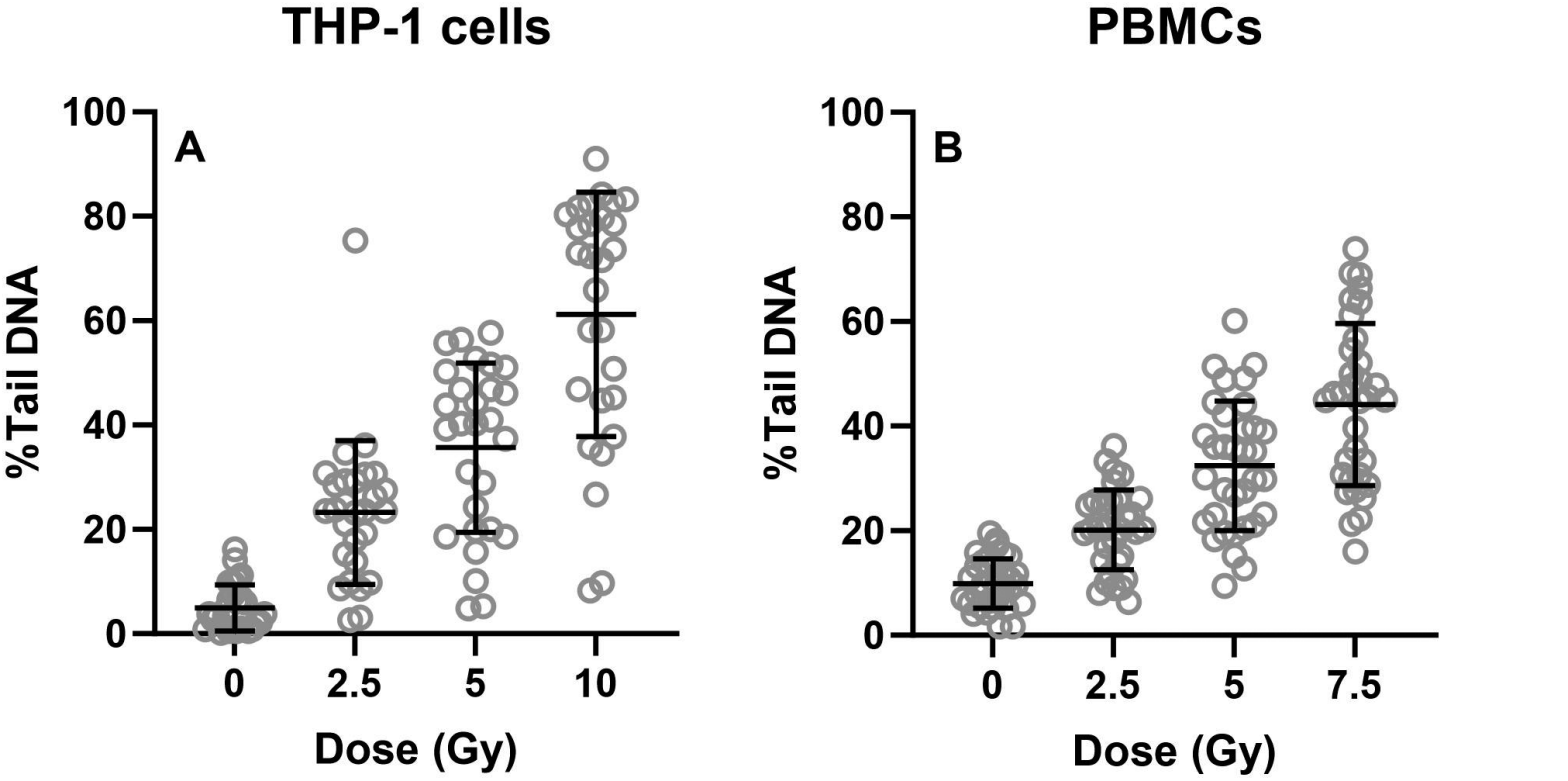
Dose-response curves from the European Comet Assay Validation Group (ECVAG) ring trials on monocytic THP-1 cells and peripheral blood mononuclear cells (PBMCs) after exposure to ionizing radiation. Identical samples were generated in a central laboratory and shipped to other laboratories for analyses. The laboratories used their own comet assay protocol in 1-3 independent experiments. Grey circles are individual results. Lines are means and standard deviations. The results have been abstracted from previously published data [41,42,45,47–49].

Another aspect of slightly increased levels of DNA migration is that the variation depends on the level of DNA damage. This is illustrated by results from the European Comet Assay Validation Group (ECVAG) ring trial where the cells were exposed to ionizing radiation in a central laboratory and batches were subsequently forwarded to different laboratories for analysis in the comet assay [41,42,45,47–49]. Figure 3 shows results from these experiments on monocytic THP-1 cells (Fig. 3A) and peripheral blood mononuclear cells (Fig. 3B). As can be seen, the variation increases proportionally with the mean level of DNA migration. It implies that the variation for small differences of DNA migration values is not the same at the ‘low-dose’ and ‘high-dose’ ends of the comet assay. In the most extreme case (Fig. 3A), unexposed cells have a mean DNA migration of 5.0% Tail DNA (Min = 0.2% and Max = 16%), whereas the highest dose (10 Gy) has a mean of 61% Tail DNA (Min = 8.3% and Max = 91%). Therefore, a slightly increased level of DNA migration will seem increasingly less biologically significant as the background level of DNA damage increases, even though net number of DNA lesions will be identical (e.g. 1–2% Tail DNA).

## Magnitude of unsystematic variation in the comet assay

Unsystematic variation in DNA migration values is largely attributed to differences in comet assay conditions [63]. In the context of an individual laboratory, variation may occur as a result of differences in procedures over time. For instance, adventitious intra-laboratory variation is introduced by uncontrolled factors such as different batches of materials, new equipment, environmental conditions, or speed in processing the samples. It can also be speculated that the electric potential in electrophoresis tanks is not stable over a period of several years. To the best of our knowledge, there are no good estimates of the impact of this kind of variability in the comet assay. However, there are reports from short-term studies (up to 1 year) where reference samples have been included in comet assay runs. These have shown relatively large variation in DNA strand breaks as demonstrated by coefficient of variation values (42%, using tail moment) [64] and oxidatively damaged DNA (52 %, using %Tail DNA) [65]. Recent results from a compilation of historical negative control data on rat liver tissue also demonstrate a relatively large variation within laboratories (variation coefficients are not reported, but the fact that original data are depicted on a logarithmic scale reflects the magnitude of variation) [66].

**Figure 4.**
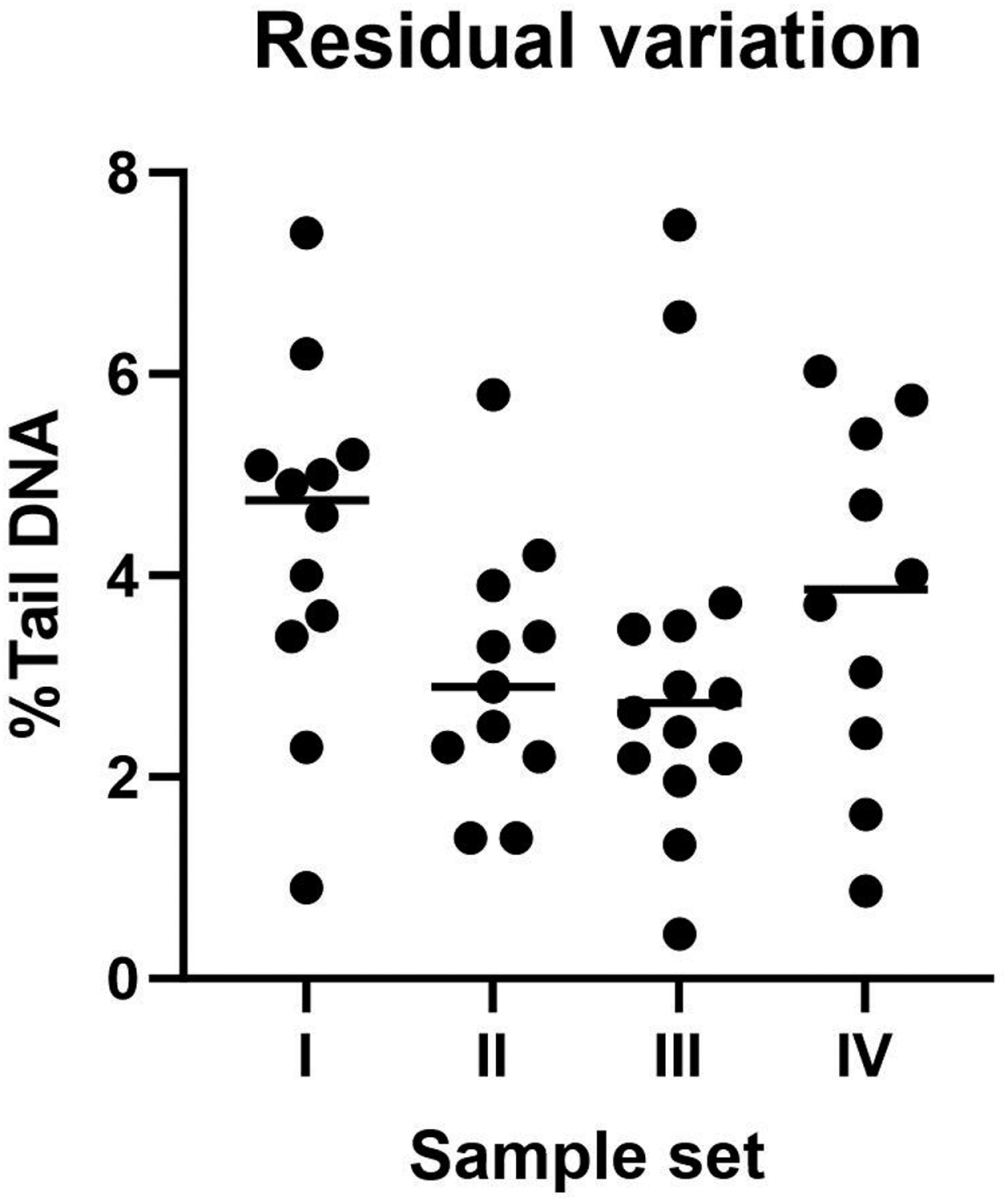
Examples of unsystematic (residual) variation in the European Comet Assay Validation Group (ECVAG) ring trial. The figure depicts the residual variation (SD_res_) from linear regression analysis of datasets of cells that have been exposed to ionizing radiation. Each symbol is the SD_res_ of linear regression analysis in one laboratory. Sample set I: three independent experiments of samples of THP-1 cells exposed 0, 2.5, 5, and 10 Gy [41]. Sample set II: three independent experiments on samples of peripheral blood mononuclear cells exposed 0, 2.5, 5 and 7.5 Gy [47]. Sample set III and IV are experiments on the same samples (peripheral blood mononuclear cells exposed 0, 2.5, 5, and 7.5 Gy) by either laboratory-specific (sample set III) or standard (sample set IV) comet assay procedures, respectively [45].

The ECVAG ring-trials are a good source of information on unsystematic variation that is seen in different laboratories because the same batch of samples were analysed by different laboratories. As these estimates are based on statistical analyses, systematic variation refers to the effect of the independent variable (i.e. predictor) on the outcome, whereas the unsystematic variation is variance that cannot be explained by the variation of the predictors. In other words, it is the variation that is left in the dataset, when the variance of all predictors has been accounted for (i.e. it is the residue of the total variance or residual variation). Figure 4 depicts the unsystematic (residual) variation of cells exposed to ionizing radiation in studies from the ECVAG ring trial [41,45]. A central laboratory made the samples and shipped them to other laboratories as cryopreserved vials. The unsystematic variation in each laboratory is based on three independent experiments with monocytic (THP-1) cells exposed to 0, 2.5, 5, and 10 Gy (sample set I) [41], three independent experiments with peripheral blood mononuclear cells exposed to 0, 2.5, 5 and 7.5 Gy (sample set II) [47], and one experiment on sample set II using either laboratory-specific (sample set III) or standard comet assay procedures (sample set IV) [45]. As can be seen, the average level of residual variation is approximately 4% Tail DNA in sets of samples with a wide range of DNA migration. In principle, it means that a difference of 4% Tail DNA could be due to assay variation in the average laboratory.

## The impact of hedgehog comets on central tendencies

In regard to calculations of the central tendency of the distribution of comets, a recurrent topic is whether attention should be paid to a subset of hedgehog comets (i.e. comets with a small head and a large tail). A small increase in DNA migration in treated cells compared to untreated ones can be produced by the increase in migration of the majority of the comets or a few comets with anomalously high % Tail DNA. Using means or medians as central tendencies will not affect the net difference between groups in the case where exposure to a genotoxic agent causes a shift in the whole distribution of comets. However, there might also be heterogeneity in the exposure, which in theory should give rise to a distribution of comets where some are damaged and others are not. The Organisation for Economic Co-operation and Development guideline for performing the *in vivo* comet assay states that the frequency of hedgehog comets (i.e. comets with a small head and a large tail) should be separately documented [67]—though they might simply represent comets at the upper limit of detection by this very sensitive assay. The presence of hedgehog comets in the results has an effect on the mean level of DNA migration. This can be illustrated by a theoretical example; of 50 comets scored, 49 comets have 1% Tail DNA (i.e. little damage) and one comet has 80% Tail DNA (i.e. a hedgehog comet), giving an average of 2.6% Tail DNA. Thus, the net difference would be 1.6% Tail DNA when comparing the DNA migration to a sample with 50 comets with 1% Tail DNA. Using the median rather than the mean gives identical results for the two samples (i.e. 1% Tail DNA). In this hypothetical example, the main question is whether the small difference in the central tendency has biological significance. It can be argued that it is relevant information because cancer in principle can develop by *chance* from clonal selection of a single cell. However, there is also a *chance* that a hedgehog comet may have been formed by cytotoxicity and apoptosis [68]. Such cells would not be expected to be sufficiently viable to develop into tumours. In addition, it has been demonstrated that hedgehog comets do not always represent early apoptotic cells; cell culture experiments have shown that DNA strand breaks in cells that form hedgehog comets are repaired [69]. Thus, hedgehog comets do not produce a straightforward answer to the significance of small differences in DNA migration values.

## General discussion and conclusions

In the present review, we have aimed at producing a balanced description of pros and cons of the notion that small differences in DNA migration values by the comet assay are statistically and biologically significant. Table 3 summarizes the pros and cons of the biological significance of slight differences in DNA damage measured by the comet assay.

**Table 3.** Weight for/against biological significance of slightly increased DNA migration level in the comet assay^a^

*Not favouring biological significance of slightly increased DNA migration level (cons)*
Intra-laboratory (day-to-day) variation is larger than slight DNA migration
The unsystematic (residual) variation is larger than slight DNA migration
Can be the effect of only few comets due to heterogeneity in the agarose gel matrix (i.e. hotspots with large mesh size)
Slight differences in DNA migration cannot be discerned visually when scoring comets (i.e. concern about the difference being real if you cannot see it)

*Favouring biological significance of slightly increased DNA migration level (pros)*
Slight DNA migration increase corresponds to hundreds of lesions (when using calibration with ionizing radiation)
Statistical analysis is based on paired samples (e.g. pre- and post-exposure, or unexposed and exposed, in the same experiment)
It may only require one damaged cell to create a cancer (e.g. a cell with many DNA lesions will gives rise to a hedgehog comet, which is reflected in a slightly increased mean level of DNA migration)

^a^In the table, ‘slight’ refers to a difference in DNA migration in the order of a few %Tail DNA units increase.

The cons pertain mainly to the variation in the comet assay, namely unsystematic variation larger than the variation that would be regarded as a slightly increased level of DNA migration. Moreover, slight differences in DNA migration might not be discerned visually when scoring comets (raising concern about the difference being real if you cannot see it). There may be relatively large variation in the comet distribution and it is not clear if this is due for example to heterogeneity in the agarose gel matrix (i.e. hotspots with large mesh size) or to a subset of highly damaged comets.

There are also certain facts in favour of the biological significance of slight differences in DNA damage measured by the comet assay (Table 3). Studies on ionizing radiation in cell cultures indicate that the comet assay can detect the difference in samples with relatively few lesions. For %Tail DNA as comet descriptor, studies have reported statistically significant effects at net differences of 0.8–3.1% Tail DNA [25–27,29–31]. Analysis of low-dose exposure studies has shown statistically significant increases are obtained by exposure to 0.3 Gy (Table 1). This indicates that the comet assay can distinguish between samples that have a difference of about 350 lesions per diploid cell. However, this is a conservative estimate and the lower boundary of the threshold dose may be 0.05 Gy, corresponding to a net difference as low as approximately 50 lesions per diploid cell. In perspective, clinically relevant functional impairment of tissues occurs at doses higher than 0.01 Gy. Acute radiation sickness is observed at doses higher than 0.07 Gy (bone marrow syndrome). Increased risks of cancer can be detected at 0.01 Sv and the risk increases at 5.5% per Sv [10]. Thus, a dose of 0.3 Gy (or 0.3 Sv) of ionizing radiation is expected to be associated with the development of cancer in 165 out of 10.000 subjects. Thus, it seems that 0.05–0.30 Gy will induce a number of lesions that can increase the risk of cancer (i.e. the cancer can start in a cell that has received this dose of radiation). As such, it could be suggested that an increase of 50–350 lesions per diploid cells is biologically significant. Moreover, only one cell is in principle needed to create cancer and one such hedgehog comet with a relatively large number of DNA lesions corresponds to a slightly increased mean of the comet distribution.

Another item that favours biological significance of slightly increased levels of DNA migration is that the intra-experiment variation is usually low in the comet assay and most analyses are intentionally (or by habit) designed to mitigate the effect of inter-assay variation. For instance, running samples from unexposed and exposed cells/animals, or samples from pre- and post-exposure time points, in the same comet assay experiment decreases the proportion of unsystematic (residual) variation. There are also benefits from designing the comet assay experiment cleverly; pairing samples (or nesting exposures in subjects) should increase the statistical power of the study.

In conclusion, weighting pros and cons does not produce a definitive answer to the biological significance of slightly increased levels of DNA migration in the comet assay. A dose-response will support the biological significance of the findings, but, in many situations, it is impossible to obtain dose–response relationships because high exposures do not occur (e.g. in human biomonitoring studies). Nevertheless, in favour of a biological significance for small differences in DNA migration is the fact that it can correspond to hundreds of lesions per cell, which may give rise to mutations or clastogenic effects if not repaired. As previously mentioned, cell culture studies on ionizing radiation have shown statistically significant effects when the net increase of DNA migration is 0.3–3.1% Tail DNA. Under optimal assay conditions, significant effects are observed at doses around 0.30 Gy, corresponding to approximately 350 lesions per diploid cell. However, the same dose of ionizing radiation can give rise to different levels of DNA migration (i.e. 0.7–7.8% Tail DNA per Gy) in different studies. Thus, the results show that even a small statistically significant difference in DNA migration has biological significance within the same experiment, but comparisons of DNA migration values between studies have limited biological implications.

## Supplementary data

Supplementary data is available at *Mutagenesis* Online.

geaf004_suppl_Supplementary_Materials

## Data Availability

All data shown in Figures 2, 3, and 4 were published in the original papers (see the figure legends) and are available upon reasonable request.
