# Supplementary material for: Slightly increased level of DNA migration in the comet assay: does statistical significance equal biological significance?
Source: Mutagenesis. 2025 Feb 18;40(2):99–110. doi: 10.1093/mutage/geaf004 (PMC12022222; doi:10.1093/mutage/geaf004)
Supplement: geaf004_suppl_Supplementary_Materials [file geaf004_suppl_supplementary_materials.pdf]

Supplement

Slightly increased level of DNA migration in the comet assay: does statistical significance equal biological significance?

Peter Møller[1], Andrew Collins[2,3], Adriana Rodriguez-Garraus[4], Sabine A.S. Langie[5], Roger Godschalk[5], Amaya Azqueta[4]*

[1]Department of Public Health, Section of Environmental Health, University of Copenhagen, Øster Farimagsgade 5A, DK-1014 Copenhagen K, Denmark.

[2]Department of Nutrition, University of Oslo, Oslo, Norway.

[3]NorGenotech AS, Oslo, Norway.

[4]Department of Pharmaceutical Science, School of Pharmacy and Nutrition. University of Navarra, C/Irunlarrea 1, 31009 Pamplona, Spain.

[5]Department of Pharmacology & Toxicology, School for Nutrition and Translational Research in Metabolism (NUTRIM), Maastricht University, Maastricht, The Netherlands.

Content

Page 2. Calibration of the comet assay with ionizing radiation.

Page 3. Ionizing radiation as genotoxic agent in comet assay experiments.

Page 5. Systematic review of studies on ionizing radiation in mammalian cells

Calibration of the comet assay with ionizing radiation

Ionizing radiation has been instrumental for the calibration of the comet assay and other techniques that detect DNA strand breaks. By "calibration" means that the primary comet descriptor is transformed to a number of lesions relative to the number of unaltered nucleobases, base pairs, or per diploid cell. **Supplementary Figure S1** depicts the procedure for generating a calibration curve in the comet assay (1,2). Samples of cells exposed to ionizing radiation are analysed in the comet assay. The results are depicted as dose (y-axis) per comet descriptor (x-axis), which produces Gy-equivalents per unit comet descriptor (i.e. the slope of linear regression). Subsequently, two assumptions are needed to produce the relationship between the comet descriptor and number of lesions in DNA. It is necessary to know 1) the relationship between the dose of ionizing radiation and yield of strand breaks in DNA and 2) the amount of DNA in mammalian cells or the molecular weight of a base pair.

The United Nations Scientific Committee on the Effects of Atomic Radiation (UNSCEAR) reports an average yield of 1000 single strand breaks, 500 base lesions and 40 double strand breaks per Gy of ionizing radiation per mammalian cell (3). This estimate originates mainly from experiments on sedimentation of DNA from irradiated cells in either alkaline or neutral sucrose gradients. This technique was used in the 1960s to measure genotoxic effects of ionizing radiation in species with low DNA content, whereas measuring the higher molecular weight of DNA in mammalian cells presented technical problems (4). In the alkaline sucrose sedimentation assay, radioactively labelled cells are lysed on top a sucrose gradient and the DNA is then separated by centrifugation. The sedimentation rate depends on the molecular weight of DNA; it is converted to DNA strand breaks per mass of DNA by comparing with the sedimentation rate of a DNA molecular weight marker. This technique was used to calibrate the alkaline elution and alkaline unwinding assays, which were developed in the 1970s and widely used before the comet assay became the method of choice for detection of DNA strand breaks (5). It is information from the calibration of these assays that has been used for the comet assay. For calibration of the alkaline elution assay, L1210 cells were irradiated with 10-100 Gy of X-rays and analysed by alkaline sucrose sedimentation using strong alkali condition (100 to 300 mM NaOH) (6). This yielded 0.27 breaks per 10^9 Dalton DNA per Gy (6). A similar estimate (0.31 breaks per 10^9 Dalton DNA per Gy) was used for calibration of the alkaline unwinding assay,

where the alkaline sucrose sedimentation was done using Chinese hamster cells and 20 mM NaOH (corresponding to pH = 12.3) (7). Using the average (0.29 breaks per 10^9 Dalton DNA per Gy) and assuming that mammalian cells have 4 x 10^{12} Dalton DNA, ionizing radiation produces approximately 1160 strand breaks per Gy in mammalian cells. This estimate was used by ECVAG to assess inter-laboratory differences in DNA damage levels (8).

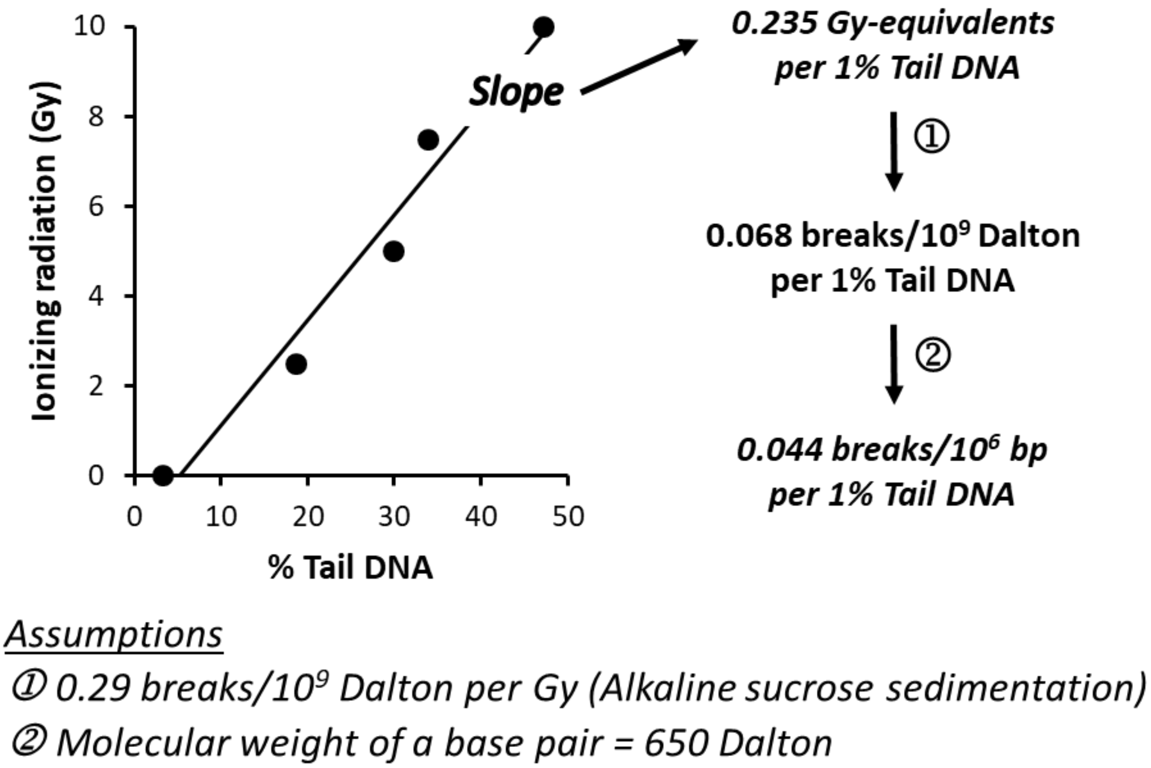

Supplementary Figure S1. Principle of ionizing radiation calibration curve in the comet assay. The dose of ionizing radiation is plotted as the function of DNA migration (e.g. %Tail DNA) and the slope is obtained as Gy-equivalents per unit DNA migration. Subsequently two assumptions leads to the final conversion factor: 1) that there is a relationship between the dose of ionizing radiation and yield of DNA strand breaks, and 2) the molecular weight of an average base pair is constant. The figure is inspired and modified from previously published presentations of comet assay calibration curves (1,2). Results on the graph stem from a calibration curve experiment at University of Copenhagen, Denmark.

Ionizing radiation as genotoxic agent in comet assay experiments

Ionizing radiation is generally categorised as according to linear energy transfer (LET) as either high-LET (e.g. alpha particles, protons and neutrons) or low-LET (e.g.

photons, electrons). LET refers to the penetration of rays in matter such as air or tissues. High-LET are large/heavy particles that move at high speed and cause damage by collision with cellular biomolecules. On the other hand, only a small portion of low-LET rays interact with molecules and they penetrate far in air or tissues. Thus, low-LET radiation has small energy transfer per unit length (implicit in the term 'low Linear Energy Transfer'). X-rays and gamma rays are low-LET radiation. X-rays are produced by rearrangement of electrons within the atom shell after nuclei have been hit by fast-moving electrons, whereas gamma rays are emitted by radioactive decay of atomic nuclei. X-rays and gamma rays inflict the same level of biological effect (i.e. their radiation weighting factor is equal to 1) (9).

The absorbed dose unit of ionizing radiation is Gray (Gy), which is the amount of energy (J) per mass (kg) of tissue. Older literature may report the absorbed dose in rads (the conversion factor is 1 rad = 0.01 Gy). In health physics, it is common to use Sievert (Sv) as equivalent (i.e. tissue-specific) or effective (i.e. whole-body) dose to assess the probability of stochastic health effects. The dose in Gy and Sv is identical in the special case of whole-body exposure to low-LET radiation. In the review, we distinguish between studies that have used low-dose (≤ 1 Gy) and high-dose (≥ 1 Gy) of ionizing radiation. This is mainly due to the fact that comet assay studies on ionizing radiation seem to fall into these categories. Studies on low-dose ionizing radiation tend to have adjusted the comet assay conditions to increase the sensitivity of the assay by adjusting steps in the protocol that favour the formation of longer comets such as increased electrophoresis time. On the other hand, studies with high maximal dose may use assay conditions that do not lead to saturation of the assay. There is no biological reason for segregating low-LET radiation as above or below 1 Gy. In perspective, clinically relevant functional impairment of tissues occurs at doses higher than 0.01 Gy. Acute radiation sickness is observed at doses higher than 0.07 Gy (bone marrow syndrome). Increased risks of cancer can be detected at 0.01 Sv and the risk increases at 5.5% per Sv (9). Thus, a dose of 1 Gy (or 1 Sv) of low-LET ionizing radiation is expected to be associated with development of cancer in 5.5 out of hundred subjects.

The DNA-damaging effect of low-LET ionising radiation occurs by direct physical interaction of photons and indirectly by secondary radical species (formed e.g. by radiolysis of water molecules). The DNA-damaging effects of low-LET ionizing radiation are mainly strand breaks and oxidized nucleobases and approximately 60% of

the lesions occur in clusters in DNA where the beam of photons have passed (9). This differs from the oxidation damage caused by reactive chemical radicals, which is randomly distributed in DNA (9).

Systematic review of studies on ionizing radiation in mammalian cells

We have carried out a systematic review of studies on associations between exposure to ionizing radiation and DNA strand breaks in mammalian cells, measured by the alkaline comet assay. PubMed was used for the search, using this string of keywords: “comet assay" AND ("ionizing radiation" OR "gamma ray" OR “gamma rays” OR “X-ray” OR "X-rays"). The search was conducted in January 2024. This produced 1143 papers, which were triaged for relevance by a tiered process with assessment of the title first and subsequently the abstract. Twenty-nine studies from the PubMed search are included in this review. Another 17 studies were identified by other means than the PubMed search, including the authors’ knowledge of a particular study (especially in the literature predating 1995), authors’ own publications, reference lists of studies that were identified in the PubMed search, and Google search using the same keywords as the PubMed search.

We have restricted the inclusion of papers to studies on gamma rays and X-rays (excluding soft X-rays as these have relatively low energy). Inclusion criteria for studies in the review were: use of the alkaline version of the comet assay (10); sufficient description of the experimental procedure (11); and use of more than one dose of ionizing radiation in mammalian cells.

In the review, studies are segregated into low-dose (≤ 1 Gy) and high-dose (≥ 1 Gy) exposure to ionizing radiation. The dose of 1 Gy is included in both of the groups. The segregation is mainly due to the fact that comet assay studies on ionizing radiation seem to fall into these categories. Studies on low-dose ionizing radiation tend to have adjusted the comet assay conditions to increase the sensitivity of the assay by altering steps in the protocol that favour the formation of longer comets, such as increased electrophoresis time. On the other hand, studies with high maximal dose may use assay conditions that do not lead to saturation of the assay. There is no biological reason for segregating ionizing radiation as above or below 1 Gy. Certain, studies have included doses below and above 1 Gy and these are included in both groups (12-16). Results on

%Tail DNA, tail length, tail moment and visual score comet descriptors are included in the review, although results as %Tail DNA have been given highest priority in descriptions of the effect size because it is considered to be the preferred comet descriptor (17).

Statistical analysis has been used to assess the influence of agarose concentration and electrophoresis conditions on the slope of dose-response curves in studies with high-dose exposure to ionizing radiation (≥1 Gy). Relationships between doses of ionizing radiation and DNA migration in the comet assay are based on linear mixed regression with agarose density (%), electrophoresis time (min) and field strength (V/cm) as predictors, and studies as categorical (absorbed) factor. The studies encompass reports where the effect of different comet assay conditions has been examined in the same study or studies with different assay conditions. Two studies from the same laboratory have been excluded as outliers from the statistical analysis because they have a high electric potential and a relatively low induction of DNA strand breaks per unit dose of ionizing radiation (18,19).

A relatively large number of the low-dose exposure studies (≤1 Gy) have assessed DNA migration as tail length (7 out of 21), tail moment (6 out of 21), visual score (1 out of 21) or comet length (1 out of 21). These descriptors cannot be compared in terms of DNA migration levels. Therefore, we have applied an analysis to assess the dose of ionizing radiation where a statistically significant increase of comet descriptors occurs in the individual studies (using χ^2-test).

Indicators of variation in the review include standard deviation (SD), standard error of the mean (SEM), 95% confidence interval (95% CI) and coefficient of variation (CV). In light of the relatively large variability in comet assay results, we have rounded off estimates of number of lesions to the nearest fifty or hundred value.

References

1. Møller, P., Cooke, M.S., Collins, A., Olinski, R., Rozalski, R., and Loft, S. (2012) Harmonising measurements of 8-oxo-7,8-dihydro-2'-deoxyguanosine in cellular DNA and urine. *Free Radic. Res*, **46**, 541-553.
2. Møller, P. (2022) Measurement of oxidatively damaged DNA in mammalian cells using the comet assay: Reflections on validity, reliability and variability. *Mutat Res Genet Toxicol Environ Mutagen*, **873**, 503423.
3. UNSCEAR (2000) United Nations Scientific Committee on the Effects of Atmic Radiation. UNSCEAR 2000 Report to the Genral Assembly, with Scientific Annexes. Sources and effects of ionizing radiation. Volume II: Effects.

4. Moroson, H., and Furlan, M. (1970) An improvement in alkaline sucrose density gradient sedimentation of mammalian cell DNA. *Radiat Res*, **44**, 713-726.
5. Møller, P. (2018) The comet assay: ready for 30 more years. *Mutagenesis*, **33**, 1-7.
6. Kohn, K.W., Erickson, L.C., Ewig, R.A.G., and Friedman, C.A. (1976) Fractionation of DNA from mammalian cells by alkaline elution. *Biochemistry*, **15**, 4629-4637.
7. Ahnström, G., and Erixon, K. (1981) Measurement of strand breaks by alkaline denaturation and hydroxyapatite chromatography. In Friedberg, E.C. and Hanawalt, P.C. (eds.), *DNA repair: A Laboratory Manual Research Procedures*. Marcel Dekker, New York, pp. 403-418.
8. Møller, P., Möller, L., Godschalk, R.W., and Jones, G.D. (2010) Assessment and reduction of comet assay variation in relation to DNA damage: studies from the European Comet Assay Validation Group. *Mutagenesis*, **25**, 109-111.
9. ICRP (2007) The 2007 Recommendations of the International Commission on Radiological Protection. ICRP publication 103. *Ann ICRP*, **37**, 1-332.
10. Collins, A., Møller, P., Gajski, G., Vodenkova, S., Abdulwahed, A., Anderson, D., Bankoglu, E.E., Bonassi, S., Boutet-Robinet, E., Brunborg, G., Chao, C., Cooke, M.S., Costa, C., Costa, S., Dhawan, A., de Lapuente, J., Del Bo', C., Dubus, J., Dusinska, M., Duthie, S.J., Yamani, N.E., Engelward, B., Gaivao, I., Giovannelli, L., Godschalk, R., Guilherme, S., Gutzkow, K.B., Habas, K., Hernandez, A., Herrero, O., Isidori, M., Jha, A.N., Knasmuller, S., Kooter, I.M., Koppen, G., Kruszewski, M., Ladeira, C., Laffon, B., Larramendy, M., Hegarat, L.L., Lewies, A., Lewinska, A., Liwszyc, G.E., de Cerain, A.L., Manjanatha, M., Marcos, R., Milic, M., de Andrade, V.M., Moretti, M., Muruzabal, D., Novak, M., Oliveira, R., Olsen, A.K., Owiti, N., Pacheco, M., Pandey, A.K., Pfuhler, S., Pourrut, B., Reisinger, K., Rojas, E., Runden-Pran, E., Sanz-Serrano, J., Shaposhnikov, S., Sipinen, V., Smeets, K., Stopper, H., Teixeira, J.P., Valdiglesias, V., Valverde, M., van Acker, F., van Schooten, F.J., Vasquez, M., Wentzel, J.F., Wnuk, M., Wouters, A., Zegura, B., Zikmund, T., Langie, S.A.S., and Azqueta, A. (2023) Measuring DNA modifications with the comet assay: a compendium of protocols. *Nat Protoc*, **18**, 929-989.
11. Møller, P., Azqueta, A., Boutet-Robinet, E., Koppen, G., Bonassi, S., Milic, M., Gajski, G., Costa, S., Teixeira, J.P., Costa, P.C., Dusinska, M., Godschalk, R., Brunborg, G., Gutzkow, K.B., Giovannelli, L., Cooke, M.S., Richling, E., Laffon, B., Valdiglesias, V., Basaran, N., Del Bo', C., Zegura, B., Novak, M., Stopper, H., Vodicka, P., Vodenkova, S., de Andrade, V.M., Sramkova, M., Gabelova, A., Collins, A., and Langie, S.A.S. (2020) Minimum Information for Reporting on the Comet Assay (MIRCA): recommendations for describing comet assay procedures and results. *Nat. Protoc,* **15**, 3817-3826.
12. Kennedy, E.K., McNamee, J.P., Prud'homme Lalonde, L., Jones, T., and Wilkinson, D. (2012) Acellular comet assay: a tool for assessing variables influencing the alkaline comet assay. *Radiat Prot Dosimetry*, **148**, 155-161.
13. Bannik, K., Rossler, U., Faus-Kessler, T., Gomolka, M., Hornhardt, S., Dalke, C., Klymenko, O., Rosemann, M., Trott, K.R., Atkinson, M., Kulka, U., and Graw, J. (2013) Are mouse lens epithelial cells more sensitive to gamma-irradiation than lymphocytes? *Radiat Environ Biophys*, **52**, 279-286.
14. Gutzkow, K.B., Langleite, T.M., Meier, S., Graupner, A., Collins, A.R., and Brunborg, G. (2013) High-throughput comet assay using 96 minigels. *Mutagenesis*, **28**, 333-340.
15. Toprani, S.M., and Das, B. (2015) Role of base excision repair genes and proteins in gamma-irradiated resting human peripheral blood mononuclear cells. *Mutagenesis*, **30**, 247-261.
16. Gomolka, M., Rossler, U., Hornhardt, S., Walsh, L., Panzer, W., and Schmid, E. (2005) Measurement of the initial levels of DNA damage in human lymphocytes induced by 29 kV X rays (mammography X rays) relative to 220 kV X rays and gamma rays. *Radiat Res*, **163**, 510-519.

17. Koppen, G., Azqueta, A., Pourrut, B., Brunborg, G., Collins, A.R., and Langie, S.A.S. (2017) The next three decades of the comet assay: a report of the 11th International Comet Assay Workshop. *Mutagenesis*, **32**, 397-408.
18. Sirota, N.P., Zhanataev, A.K., Kuznetsova, E.A., Khizhnyak, E.P., Anisina, E.A., and Durnev, A.D. (2014) Some causes of inter-laboratory variation in the results of comet assay. *Mutat. Res Genet. Toxicol Environ Mutagen*, **770**, 16-22.
19. Sirota, N., Kuznetsova, E., and Mitroshina, I. (2018) The level of DNA damage in mouse hematopoietic cells and in frog and human blood cells, as induced by the action of reactive oxygen species in vitro. *Radiat Environ Biophys*, **57**, 115-121.
